# Importance of Body Stance in Fog Droplet Collection by the Namib Desert Beetle

**DOI:** 10.3390/biomimetics4030059

**Published:** 2019-08-28

**Authors:** Unmeelan Chakrabarti, Roberto Paoli, Souvick Chatterjee, Constantine M. Megaridis

**Affiliations:** 1 Department of Mechanical and Industrial Engineering, University of Illinois at Chicago, Chicago, IL 60607, USA; 2 Computational Science Division and Leadership Computing Facility, Argonne National Laboratory, Lemont, IL 60439, USA

**Keywords:** Namib desert beetle, fog basking, computational fluid dynamics, fluid flow patterns, droplet collection efficiency

## Abstract

The fog-basking behavior of the *Onymacris unguicularis*, a beetle species living in the coastal regions of the Namibian desert, has recently caught the attention of the engineering community, as suggesting a viable biomimetic approach to address the problem of harvesting water in arid regions of the globe. Previous research has focused on observation and analysis of the beetle’s elytron properties and how these affect fog-collection rates. The head stance taken by the *Onymacris unguicularis* when fog basking is well documented. However, how this stance affects droplet collection has not been studied up to now. The present paper addresses this problem from a computational fluid dynamics perspective, where three-dimensional numerical simulations are used to characterize the fog flow properties around a simplified geometry mimicking the beetle’s body. The simulations employ two-way coupling between the gas flow and the dispersed fog phase to account for feedback effects of fog droplets on the carrier fluid (air), and assume that droplets are captured after hitting the elytron surface. The study considers several combinations of free-stream velocity and droplet volume fraction. The analysis reveals that there is a range of head-stance angles, corresponding to an inclination of the beetle between 35 deg and 45 deg with respect to the horizon, that maximizes water collection on the beetle’s back, in qualitative agreement with observations in nature and laboratory experiments. A rationale is proposed to explain this phenomenon, finding that the specific head stance corresponds to the maximum residence time of fluid particles above the beetle’s elytron surface. This, in turn, designates the maximum likelihood for water droplets to be captured in the boundary layer developing over the beetle and subsequently hit the surface where they get captured. The results reveal the importance of the fluid flow pattern around the beetle’s body in addition to the microphysical properties of the elytron when reliable predictions of the water droplet collection efficiency are sought.

## 1. Introduction

Water in the atmosphere has been an elusive freshwater resource in arid regions of the globe. In their 2001 article, Parker and Lawrence [1] suggested a biomimetic approach for harvesting water efficiently from the atmosphere. The arguments presented in that work relied on the morphology and wettability of a desert beetle’s back, both presumed to play a critical role in the water-droplet collection process. The article by Parker and Lawrence inspired numerous follow-up studies, many of them using biomimetic principles and arguments to motivate devices in the technological arena [2,3,4]. Subsequent studies showed substantially improved water collection rates on inhomogeneous-wettability surfaces, as compared to flat or uniformly-wettable surfaces [5,6]. Similar effects were also observed in studies of dew condensation on desert beetles [7].

**Figure 1:**
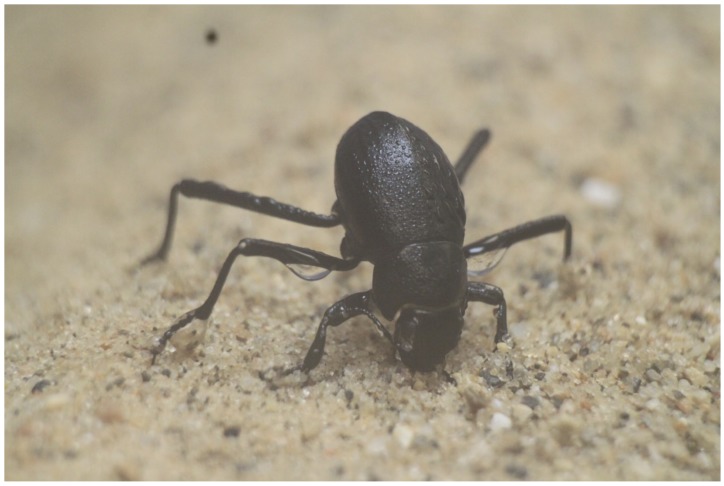
Photograph of an *Onymacris unguicularis* beetle in the basking position, showing the typical downward head stance, which allows its legs and the rest of its body to collect water. The photograph was taken in our laboratory during experiments with live specimens.

Several comparative studies that evaluated fog basking among the more than 200 Namib Desert tenebrionid species, have found only two species—both Onymacris—to be fog baskers [8]. *Onymacris unguicularis* is one of them; its dorsal surface (a.k.a. elytron) features uniform wettability [9]. Nørgaard and Dacke [9] reported that boundary layer structure may play a critical role in the fog-collection mechanism, and this particular possibility remains unexplored. Their work pointed to the importance of the boundary layer structure around the body of the beetle and its effects on the transport mechanisms under realistic fog-basking conditions. They observed that during fog basking the *O. ungucularis* beetle takes a head stance, so that the ventral side of the animal is at an angle of ∼23 deg to the horizontal. Such posture was consistently observed in experiments conducted with live beetles (see Figure 1). The significance of such a head stance from the fluid mechanics perspective is intriguing. Here, we carry out a numerical study to characterize the flow properties around a simplified geometry (semi-ellipsoid) resembling that of the *O. unguicularis* beetle. Specifically, the objective is to understand whether the posture assumed by the beetle to capture water droplets can be at least partly explained using fluid mechanical arguments, for example, is there a relation between the head stance and the three-dimensional boundary layer developing around the body, which maximizes water collection? To answer these questions, we perform numerical simulations for different inclinations of the ellipsoid that mimics the head stance of the beetle, and monitor the corresponding collection efficiency (number of water droplets collected on the beetle surface) using a three-dimensional Navier–Stokes solver with two-phase flow capabilities to track water droplets.

## 2. Methods

All simulations were carried out in OpenFOAM (open source field operation and manipulation), an open source C++ toolbox for developing numerical solvers for various problems in the field of computational fluid dynamics [10]. The OpenFOAM solver used for this study, reactingParcelFilmFoam, is based on an Eulerian–Lagrangian two-phase flow modeling for the continuous phase (carrier fluid, air) and the dispersed phase (water droplets), respectively (see OpenFOAM documentation) https://openfoam.org/resources. The two phases are coupled through a two-way coupling procedure in which the carrier fluid and the convected droplets interact with each other via drag forces. The continuous phase behavior is governed by the Navier–Stokes equations for an incompressible fluid,

(1) $$\begin{array}{ccc} \nabla \cdot u & = & 0 \end{array}$$

(2) $$\begin{array}{ccc} \frac{\partial u}{\partial t}+u\left(\nabla u\right) & = & -\frac{1}{\rho }\nabla p+\nu \nabla^{2}u+g+F_{D}, \end{array}$$

where u,ρ,p are the fluid velocity vector, density and pressure, respectively, ν is the kinematic viscosity of the fluid, g is the gravity acceleration vector, and $F_{D}$ the local force exerted by the droplets on the fluid in the two-way coupling setting. We also evaluated the importance of this term in the calculation of the overall mass collected on the body’s surface using a one-way coupled setting (see Appendix A). Water droplets were tracked individually using the point-particle approximation and their trajectories were obtained by integrating the following set of equations

(3) $$\begin{array}{ccc} \frac{dx_{p}}{dt} & = & u_{p} \end{array}$$

(4) $$\begin{array}{ccc} m_{p}\frac{du_{p}}{dt} & = & {F_{d}}_{p}+m_{p}g, \end{array}$$

where $x_{p}$ is the droplet position vector, $u_{p}$ the droplet velocity, $m_{p}=\rho_{p}\pi d_{p}^{3}/6$ the droplet mass, $\rho_{p}$ and $d_{p}$ are the water density and droplet diameter, respectively, while ${F_{d}}_{p}$ is the drag force exerted by the fluid on the droplet, which can be expressed as

(5) $${F_{d}}_{p}=-\frac{1}{2}\rho \left|v_{p}\right|v_{p}S_{p}\;{C_{d}}_{p},$$

where $v_{p}\equiv u_{p}-u\left(x_{p}\right)$ is the relative velocity of the droplet with respect to the surrounding fluid, $S_{p}\equiv \pi d_{p}^{2}/4$ is the droplet cross-sectional area, while ${C_{d}}_{p}$ is the drag coefficient approximated by the Stokes drag formula, ${C_{d}}_{p}=24/Re_{p}$, where $Re_{p}\equiv d_{p}\left|v_{p}\right|/\nu$ is the Reynolds number. This assumption is justified in the present calculations by the small droplet diameter and small relative velocity, which gives $Re_{p}\leq 1$. The governing Equations (1) and (2) were solved using the PIMPLE algorithm, which is a combination of two different algorithms, PISO (pressure-implicit split-operator) and SIMPLE (semi-implicit method for pressure linked equations). During each time step, we solved a pressure equation, to enforce mass conservation, within an explicit velocity correction to satisfy momentum conservation. The module reactingParcelFilmFoam implemented in OpenFOAM was used to resolve Lagrangian particle motion and for the impingement of droplets on the surface of the body. To that end, a special external mesh region was extruded from the body’s surface. For modeling of the film layer, a thin-film approximation was used: the velocity normal to the wall was zero while, along the wall, the tangential diffusion was considered to be negligible compared to normal diffusion. The surface film flow was solved using the continuity and momentum equations. The continuity equation comprised of a source term representing the mass added to the film layer due to the incoming fog droplets ($S_{\mathrm{imp}}$)

(6) $$\frac{\partial \rho_{f}\delta_{f}}{\partial t}+\nabla \cdot \left(\rho_{f}\delta_{f}u\right)=S_{\mathrm{imp}},$$

where $\rho_{f}\equiv \rho_{\mathrm{water}}$ is the density of the film fluid and $\delta_{f}$ is the film thickness.

## 3. Results

**Figure 2:**
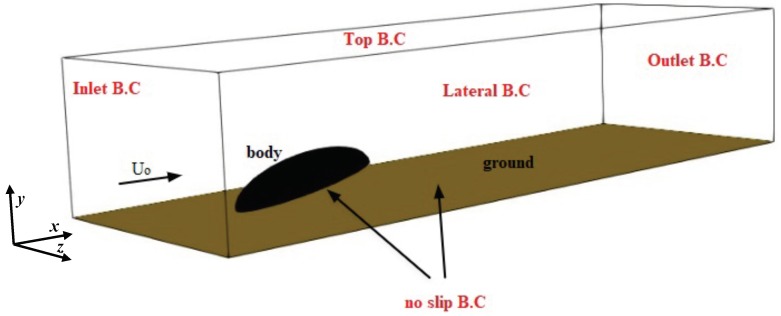
Wireframe image of the computational domain and boundary conditions (B.C.). The axes of the solid semi-ellipsoidal body are: $a_{x}=8\;\mathrm{mm}$, $a_{y}=3\;\mathrm{mm}$, and $a_{z}=3\;\mathrm{mm}$, typical of real beetles. Lateral and top B.C. are implemented as a symmetry condition, which effectively imposes no flow through these boundary surfaces. In terms of the velocity field, these conditions are equivalent to assuming wall slip, U·n=0, where n denotes the normal to each symmetry surface.

**Figure 3:**
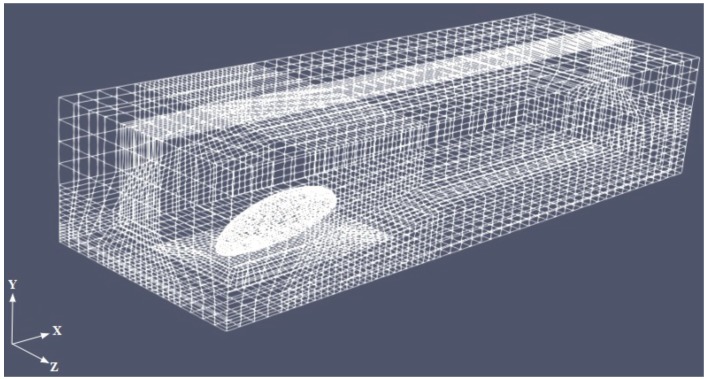
Sample computational mesh for α=30deg. The mesh is fully structured and multi-block, and features $2.5\times 10^{6}$ grid cells. The cuboid surrounding the body has dimensions: $L_{x}=20\;\mathrm{mm},L_{y}=8.5\;\mathrm{mm}$, and $L_{z}=10\;\mathrm{mm}$. For ease of visualization, only one of every five grid cells is shown in this image.

Based on the structure of the beetle’s body, a simplified geometry was chosen consisting of a semi-ellipsoid with axes of 8 mm, 3 mm, and 3 mm, in the horizontal (−x), vertical (−y) and transversal (−z) directions, respectively. The legs were not modeled due to their much lower surface area compared to the rest of the body. The semi-ellipsoid (body hereafter) was placed in a surrounding external box (cuboid shaped with side lengths $L_{x}=20\;\mathrm{mm},L_{y}=8.5\;\mathrm{mm}$, and $L_{z}=10\;\mathrm{mm}$) with inclination angle α with respect to the ground (see Figure 2 for the representative case of α=30deg). Several grids were generated for each α with the total number of cells $N=2.5\times 10^{6}$, kept constant. For each case, we performed a grid independence study to ensure the grid was fine enough to capture the flow characteristics (not shown). Figure 3 shows the mesh for α=30deg. The body is not exactly laying on the ground, but is elevated to mimic the beetle posture due to the presence of the legs (not modeled here). The boundary conditions were assigned in the form of inlet, outlet, no slip at the base wall of the box and the beetle surface, and symmetry at the lateral and top face of the box, as sketched in Figure 2. Table 1 summarizes the details of the simulations conducted in this study. In the baseline cases (runs A1–A10) the free-stream air velocity was $U_{0}=1\;m/s$ and the droplet volume fraction injected in the domain was $f_{p}=3.6\times 10^{-3}$. We also considered larger velocities and smaller volume fractions (runs B1–B9 to F1–F10 in Table 1), so as to explore the sensitivity of the results to these parameters. These values are similar to those used in our laboratory experiments with live beetles, and, in the case of velocity, they represent light to moderate breezes during foggy days in the Namib desert coastal region according to available meteorological data [11,12]. Finally, in a last set of simulations (G1–G10), we considered the influence of non-uniform droplet size distribution.

**Figure 4:**
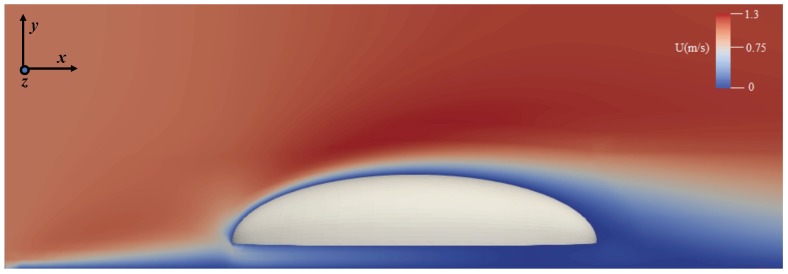
Distribution of the magnitude of fluid velocity around the body in the steady state (converged solution) for the zero incidence angle case, α=0 deg (run A1).

For each body inclination angle α, the numerical simulations were carried out in two steps. First, air was introduced at the inlet of the computational domain with uniform velocity $U_{0}$ and Equations (1) and (2) were solved with constant time step Δt until $t=t_{\mathrm{fin}}$ when the solution converged, i.e., the flow properties did not change any longer throughout the computational domain. The flow Reynolds numbers for $U_{0}=1,2.7$ and 5m/s are Re=axU0/ν=533,1439 and 2665, respectively. The first two values are low enough for the flow to be considered laminar throughout the entire domain. For the highest Re, the flow may experience incipient transition to turbulence, even though the impact of turbulence on the droplet collection on the body is likely minor—we do not expect early detachment of the boundary layer induced by low-intensity turbulence, the main effects, if any, being on the wake of the body, which is of less importance in this study. Figure 4 shows the velocity magnitude contour at $t=t_{\mathrm{fin}}$ for the representative case of zero incidence angle (run A1), and demonstrates the development of the boundary layers around the body’s surface and over the ground base. Note the confluence of the fluid as it streams inside the gap between the ground and the bottom side of the body.

**Figure 5:**
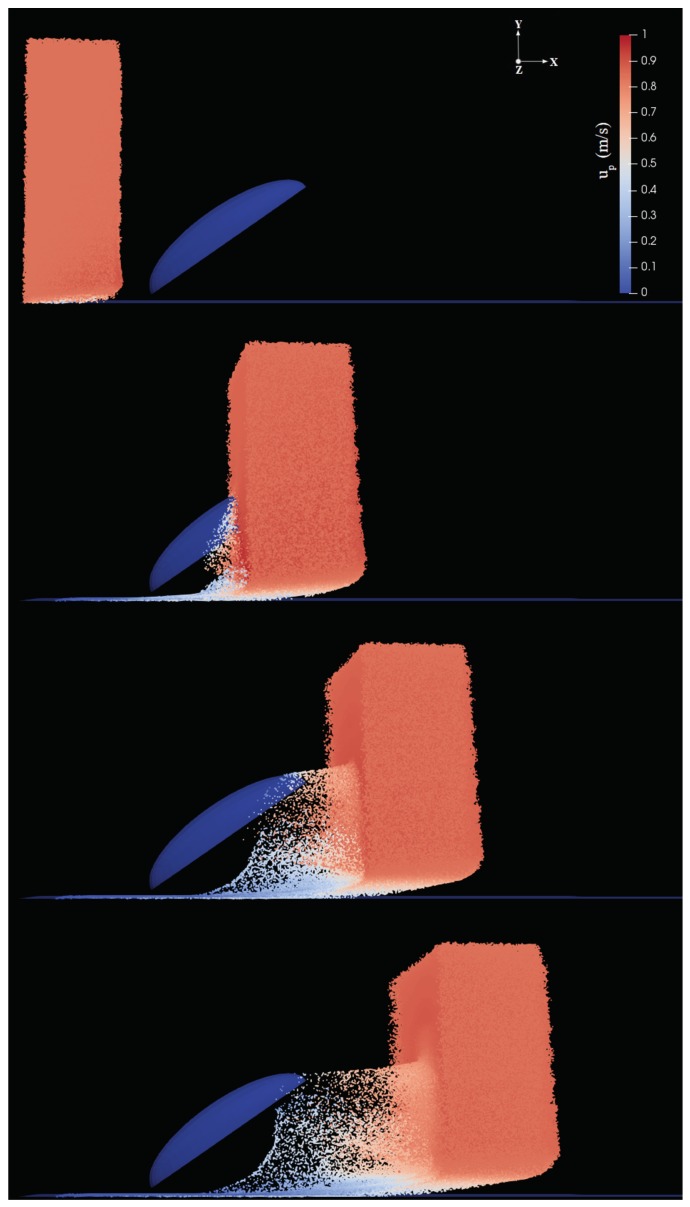
Temporal evolution of the slab of droplets injected horizontally (parallel to the ground) into the computational domain for α=40deg (run A6). Droplets are colored with the magnitude of droplet velocity ($u_{p}$ in Equation (3)). From top to bottom, panels correspond to t=3.5ms,12.5ms,17ms, and 20ms after droplets injection. An animation of this sequence is available in the Supplementary Materials (www.mdpi.com/xxx/s1).

**Figure 6:**
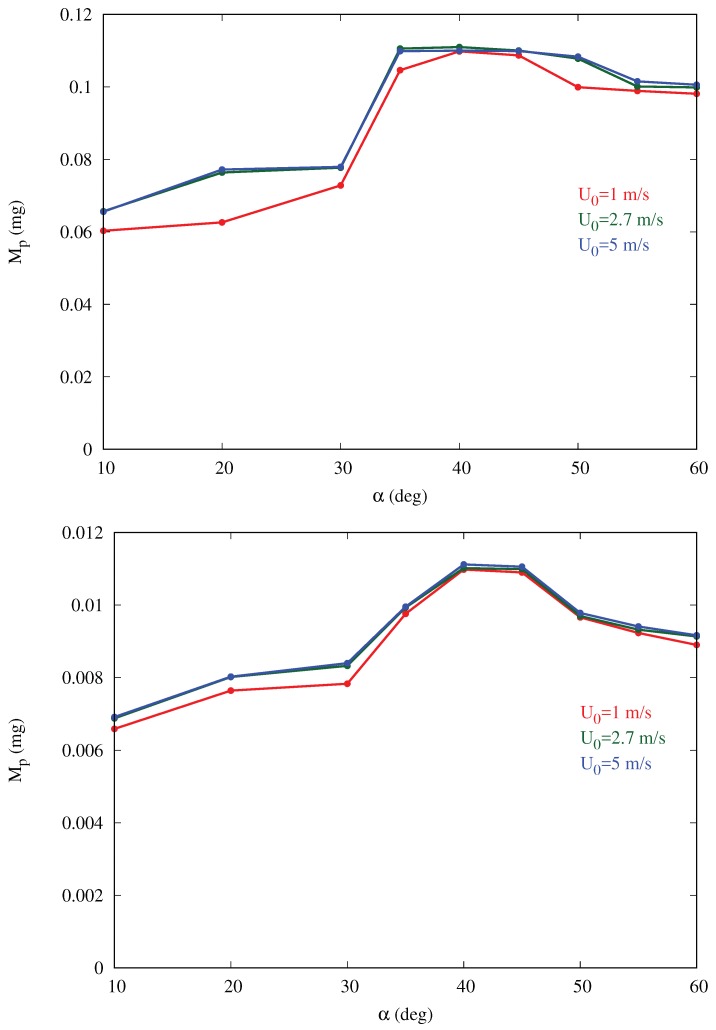
Water mass collected (as droplets) on the ellipsoidal surface vs. body inclination angle α for three different free-stream velocities $U_{0}$. The two panels correspond to different droplet volume fraction in the slab, $f_{p}$, and hence different water mass $M_{0}$ injected in the computational domain. Top panel: $f_{p}=3.6\times 10^{-3}$ and $M_{0}=1.44\;\mathrm{mg}$ (runs A2–A10, B2–B10, C2–C10 in Table 1); Bottom panel: $f_{p}=3.6\times 10^{-4}$ and $M_{0}=0.144\;\mathrm{mg}$ (runs D2–D10, E2–E10, F2–F10 in Table 1).

In the second step, Lagrangian fog droplets were injected into the environment with the same free-stream velocity of the carrier air stream (i.e., zero relative velocity with respect to the fluid). In all simulations we used the patchInjection module of OpenFOAM, where $N_{p}$ droplets were injected at the inlet. This results in a slab of droplets reaching the body, as shown for example in Figure 5. The volume of the slab is $V_{\mathrm{slab}}=L_{y}\times L_{z}\times L_{\mathrm{slab}}=400\;{\mathrm{mm}}^{3}$ where $L_{\mathrm{slab}}=4.7\;\mathrm{mm}$ is the streamwise extension of the slab. Droplets were initially homogeneously distributed inside this slab. In the uniform size distribution cases, droplets were assumed to have diameter $d_{p}=20\;\mu m$, typical of desert fog [1], the total volume and mass of water injected in the domain were thus $V_{p}=N_{p}\pi d_{p}^{3}/6$ and $M_{0}=\rho_{\mathrm{water}}V_{p}$, respectively. The droplet volume fraction in the slab was $f_{p}=V_{p}/V_{\mathrm{slab}}$ (values listed in Table 1). For the sake of verification of the two-phase flow assumptions, we compared one-way and two-way coupling simulations for the baseline cases; the outcomes were not influenced by the coupling assumption in the most critical case, i.e., larger volume fraction (see Appendix A). The droplet dynamics can be described qualitatively as follows: droplets are partly intercepted by the body, and partly either continue their trajectory with essentially the same velocity (those above the body) or penetrate the boundary layer developing on the ground (below the body). Among droplets intercepted by the body, some are captured inside the boundary layer and eventually stick to the surface, while some are able to escape (never contacting the body) and continue their trajectory in the wake with reduced velocity (see for example Figure 5d). In order to determine the droplet collection efficiency, we calculated the ratio of the mass of droplets coming in contact with the surface of the body and the mass of particles injected into the environment, for each inclination α. The data related to this mass is obtained using the surface film modeling of OpenFOAM, Equation (6). Figure 6 shows that the mass collection is maximum at α=40deg, being about 65% more than at α=10deg, and 16% more than at α=60deg for the baseline cases. This behavior is observed for all cases in Table 1 with the peak occurring at inclination between 35 and 45deg. For a given inclination angle α, the mass collection tends to increase slightly with the free-stream velocity $U_{0}$ (converging for larger values), especially in the cases with larger volume fraction $f_{p}$. This is likely due to the fact that, if velocity increases, more droplets impact the body since they are less likely to circumvent the obstacle due to their higher momentum. In addition, a smaller fraction of droplets is trapped in the thinner boundary layer developing on the ground, without ever reaching the body.

**Figure 7:**
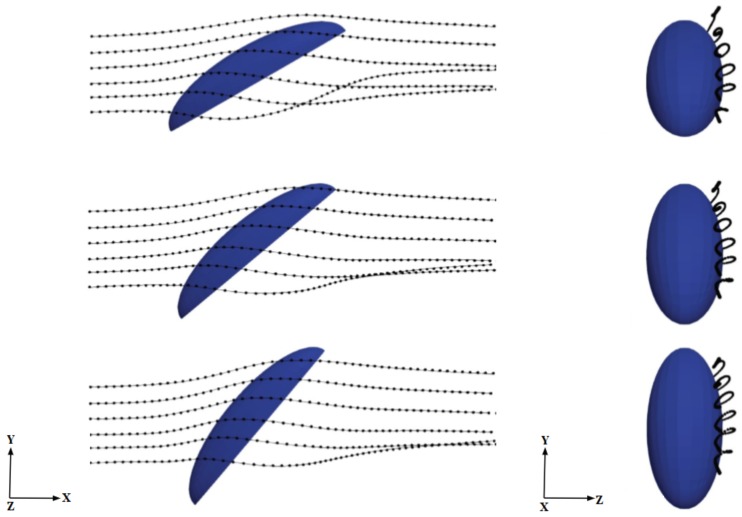
Lateral (left) and longitudinal (right) views of sample droplet trajectories. From top to bottom: α=30deg (run A4), 40 deg (run A6) and 60 deg (run A10). At their leftmost injection locations, droplets are placed on a vertical (x–y) plane at a distance of 0.87 mm from the body’s symmetry plane and the vertical separation between adjacent droplets at their injection locations is 0.2 mm.

One possible explanation for this intriguing behavior for the mass collection efficiency exhibiting a consistent peak around 40 deg is related to the three-dimensional aerodynamics of the flow around the semi-ellipsoid, which deflects the initially straight droplet trajectories. This, in turn, influences the velocity of droplets entering the boundary layer and depositing on the surface in a complex way that cannot be predicted solely on the basis of geometric arguments. In order to confirm this hypothesis, we analyzed droplet trajectories obtained by connecting the positions of selected droplets at various instants $x_{p}\left(t\right)$ as they move through the flow field (which also changes in time because of the two-way coupling). We inject particle tracers along lines located at different heights from the ground, each initially separated vertically by 0.2 mm from its nearest neighbor and all located at a distance of 0.87 mm from the central vertical symmetry plane (see Figure 7). The average arc length $S_{L}$ traced by the particles near the surface of the body and the average residence time $T_{L}$ are calculated, respectively, as

(7) $$\begin{array}{c} S_{L}=\langle \int_{s_{1}}^{s_{2}}ds_{L}\rangle \;\mathrm{and}\;T_{L}=\langle \int_{s_{1}}^{s_{2}}\frac{ds_{L}}{u(s_{L})}\rangle . \end{array}$$

**Figure 8:**
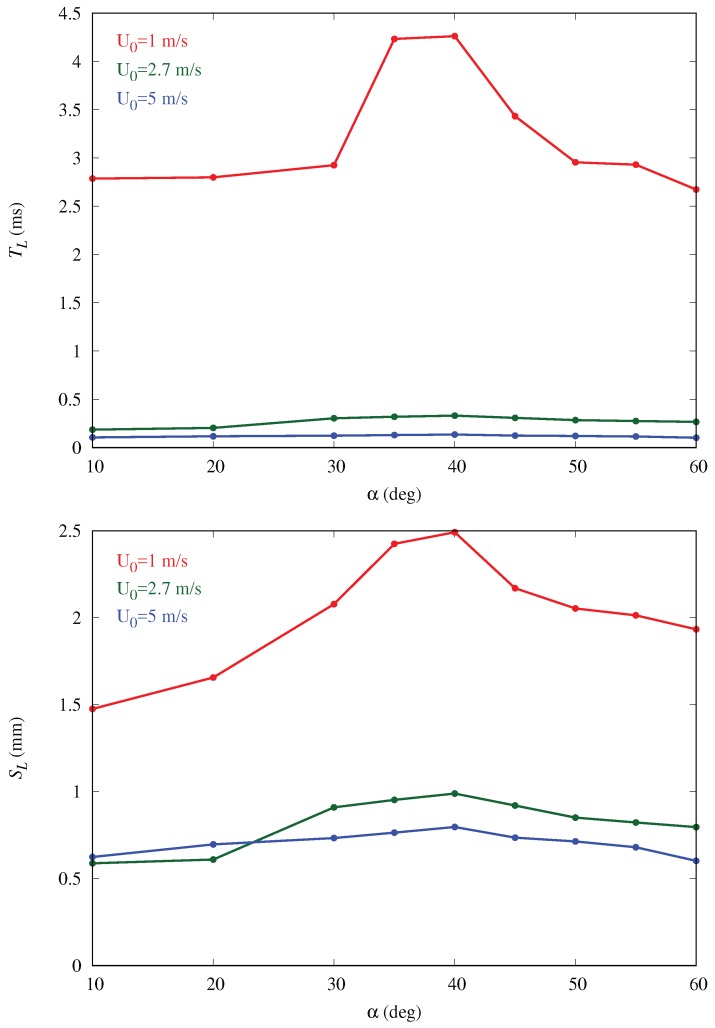
Plots of average droplet residence time (top panel) and arc length (bottom panel) for varying body inclination angles (runs A2–A10). Higher residence times are associated with higher arc lengths and higher probability for droplet capture by the body.

**Figure 9:**
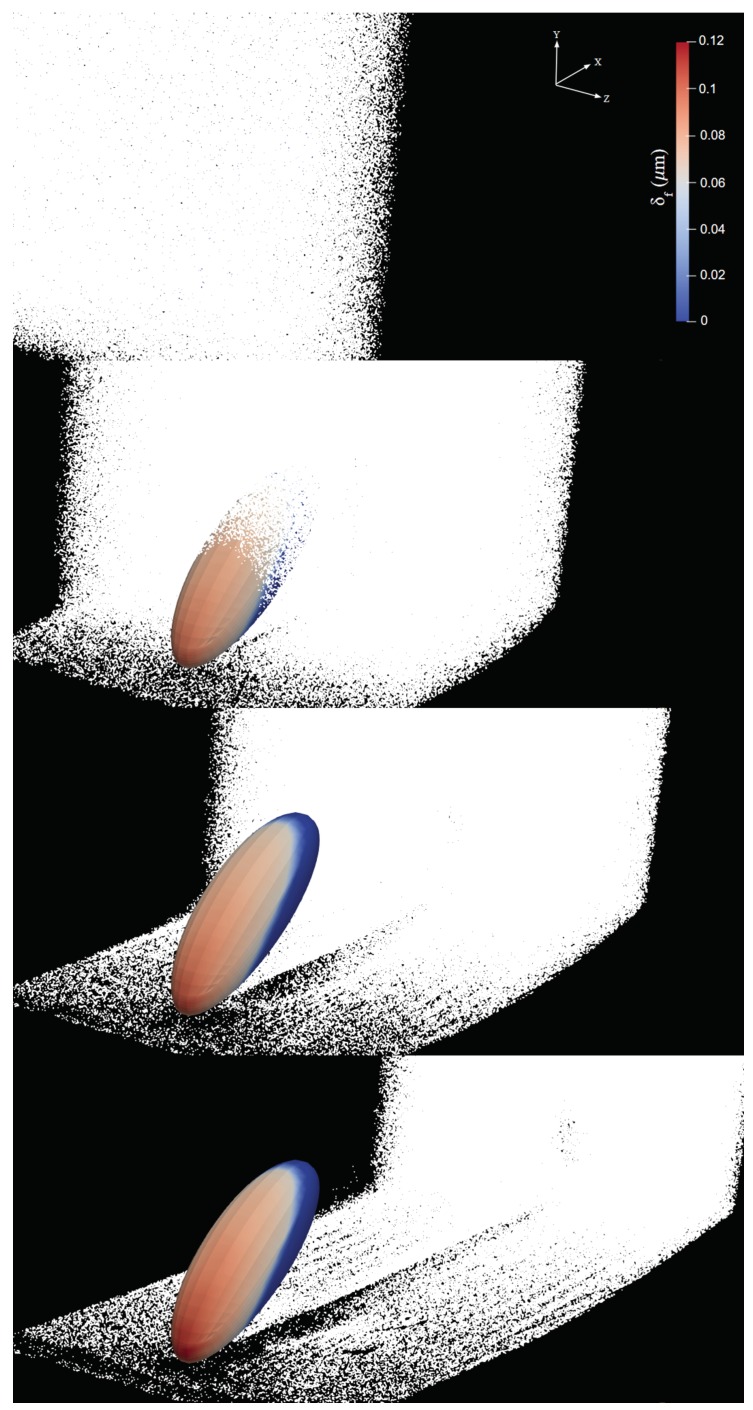
Temporal evolution of liquid-film thickness $\delta_{f}$ formed by water droplets (represented by white dots) for α=40deg (run A6). From top to bottom, panels correspond to t=3.5ms,12.5ms,17ms, and 20 ms after droplets injection. An animation is available in the Supplementary Materials (www.mdpi.com/xxx/s1).

In the above equations, $ds_{L}$ represents the differential arc-length along a given droplet trajectory with $s_{1}$ and $s_{2}$ the initial and final positions (ahead and behind the body, respectively); $u(s_{L})$ is the corresponding velocity magnitude at each location along the arc, whereas brackets denote average over the trajectories. Figure 7 shows seven trajectories at three different body inclination angles; the results are insensitive to this choice, given the smooth character of this laminar flow. Both $S_{L}$ and $T_{L}$ are plotted for varying inclinations in Figure 8. We observe that both parameters follow trends similar to those observed for mass collection efficiency with shallower variation with α for higher free-stream velocities. The rationale proposed here for this behavior is that when an approaching droplet spends more time near the surface, the probability for droplet collection on the surface rises. This is an aerodynamically-driven (rather than purely geometrically-driven) process and can only be captured by solving the full Navier–Stokes equations. In particular, the curvature of the body is crucial to explain the residence time trends. This can be appreciated from the trajectories in Figure 7 and from the three-dimensional distribution of the deposited liquid film thickness determined by Equation (6) and plotted in Figure 9 for α=40deg. The film thickness is highest around the bottom and in the middle of the body’s surface and decreases at the top as well as on the sides of the semi-ellipsoid. Increasing α would certainly increase the number of particles intercepting the body locally but would also decrease the residence time of particles around the body, thus giving droplets less time to circumvent the body and get captured in the curved boundary layer. Hence, there has to be an optimum inclination (around 40 deg in this simplified geometry) for which the two effects combine to maximize the collection efficiency. Note the difference with a purely two-dimensional geometry like a flat plate at inclination, where all trajectories would eventually impact the plate (as long as α>0) and so the maximum collection would occur for α=90 (frontal impact), based on purely geometrical reasoning.

### 3.1. Water Collection along the Surface

**Figure 10:**
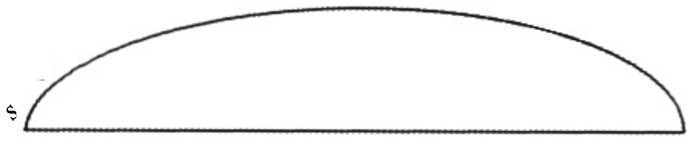
Intersection of the semi-ellipsoidal body and a plane passing through the length-wise symmetry axis at the bottom surface. An arc is thus defined on the body’s upper surface, where the water film thickness is computed. The curvilinear coordinate *s* denotes the distance from the leading edge along the upper surface.

**Figure 11:**
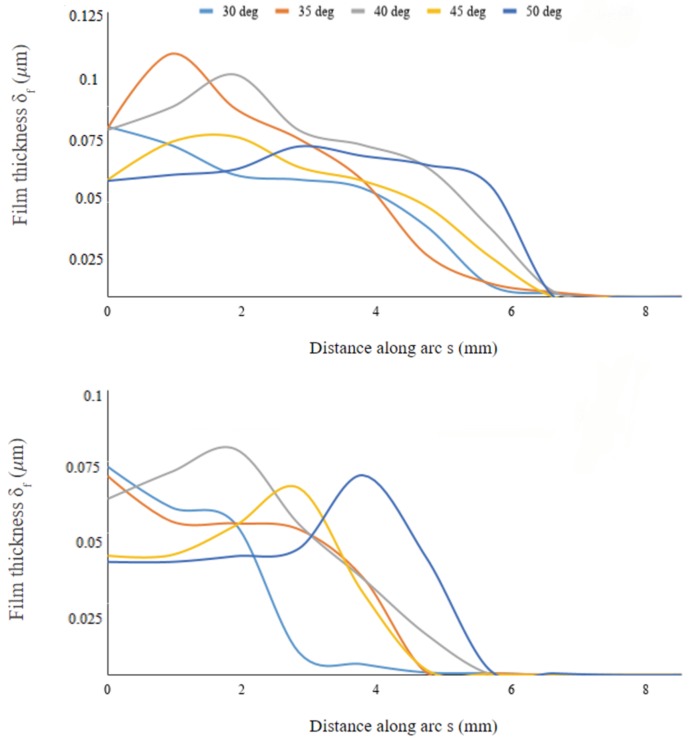
Water film thickness along selected arcs on the body’s surface and at various body inclination angles (runs A4–A8). Top panel: arc cutting through the lengthwise vertical symmetry plane of the semi-ellipsoid; Bottom panel: arc defined by the intersection of the body and an inclined plane passing through the lengthwise symmetry line at the bottom surface and turned 45 deg from the vertical symmetry plane.

To further investigate the water collection along the body surface, we determined the film thickness $\delta_{f}$ of water collected along an arc defined by the intersection of the vertical symmetry plane and the semi-ellipsoid. We did the same for another arc defined by the intersection of the body and an inclined plane passing through the lengthwise symmetry line at the bottom of the body and turned 45 deg from the symmetry plane. This procedure defines the sections along which the film thickness is measured. The data are then plotted as a function of arc length (see Figure 10) for varying inclinations of the body. Figure 11 shows the profiles to be wider on the symmetry plane compared to the eccentric plane, thus indicating that the film spreads more effectively lengthwise than laterally along the body’s surface; this is also noticeable from the snapshots of Figure 9. The film thickness peaks at different stations along the surface arc-length, depending on the inclinations angle α. On the symmetry plane, the arc-length location where $\delta_{f}$ peaks is initially located at the leading edge of the body for α≤35deg, then it moves downstream as inclination increases to α=40deg, moving back upstream for α=45deg, and downstream again for α=50deg where the distribution of $\delta_{f}$ is more uniform throughout the entire arc-length. Interestingly, this non-monotonic behavior occurs around α=35−40deg where the collected water mass is also maximum (see Figure 6). The same behavior is observed along the eccentric arc, although the displacement of the peak location is delayed compared to the displacement on the symmetry axis, with film peaking narrowly at the rear of the body when α=50deg. Hence, the geometric alignment of the beetle with respect to the incoming fog stream contributes to the high mass collection efficiency at these inclinations.

### 3.2. Results with Rosin–Rammler Droplet Distribution

**Figure 12:**
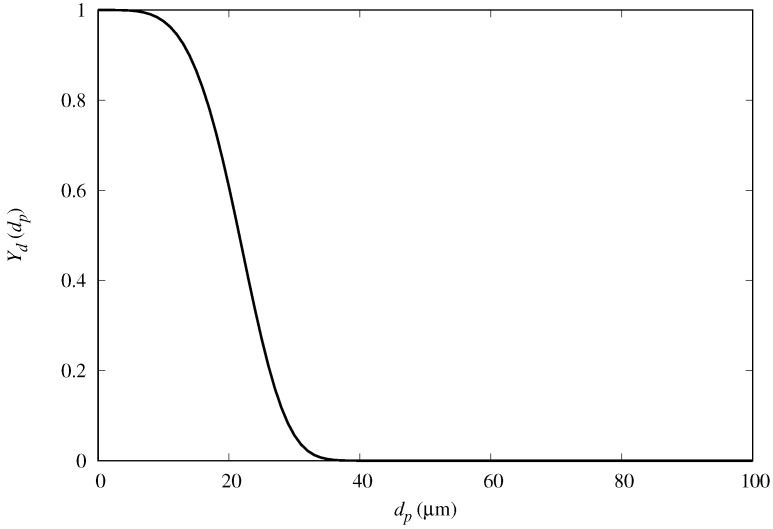
Complimentary Rosin–Rammler distribution function (Equation (8)) with $\bar{d}=23.48$ μm and n=4.32. These values were determined after analysis of the fog generated in experiments performed in our laboratory.

**Figure 13:**
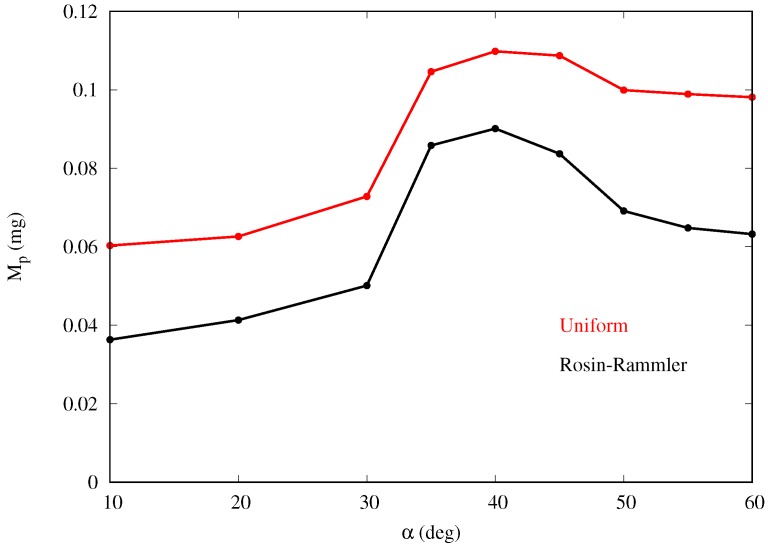
Droplet mass collection for varying body inclinations and fog droplets following the Rosin–Rammler distribution (runs G2–G10). The results obtained with uniform distribution (runs A2–A10) are also plotted for comparison.

In order to further examine the fog mass collection efficiency of the semi-ellipsoids, we compared the results for predicted mass-collection efficiency for fog with polydisperse droplet size distribution. We cast the diameter of the droplets into a Rosin–Rammler distribution, and repeat the numerical simulations with a polydispersed input flow condition in OpenFOAM (see Figure 12). Following this distribution, the fraction $Y_{d}\left(d_{p}\right)$ of droplets greater than $d_{p}$ (complimentary cumulative distribution) is expressed as

(8) $$Y_{d}\left(d_{p}\right)=e^{-{\left(d_{p}/\bar{d}\right)}^{n}},$$

where $\bar{d}$ is the mean diameter and *n* is the size distribution parameter. The values considered are $\bar{d}=23.48\;\mu m$ and n=4.32, based on fog tests performed in our laboratory with a household type humidifier. Figure 13 shows that the mass collection efficiency follows a similar trend as for the monodispersed fog cases, with maximum collection also at around α=40deg.

## 4. Conclusions

We have presented quantitative reasoning for the fog-basking abilities of a Namib desert beetle (*O. unguicularis*). With the help of numerical simulations, we examined the aerodynamic and geometric conditions that possibly motivate the beetle to bend forward during fog basking. By calculating the fog collection rates at varying geometrical alignments of the beetle’s body with respect to the horizontal ground, we determined that water collection is consistently maximized in the range 35 deg–45 deg inclination for all combinations of droplet velocities and volume fraction considered in this study (which amount to over 70 numerical simulations). These results are relevant to fog basking, as they provide evidence of a beetle stance that is optimized to capture water for given body shape and incoming flow conditions. The results of this study, the first to analyze the flow around a model beetle using CFD, are encouraging and confirm, at least qualitatively, the behavior of real beetles as observed in laboratory experiments and in their natural environment. The difference between the observed head stance angle observed experimentally by others and that predicted in the present work for maximum fog collection could be attributed to the simplified assumptions used in this study. First, the microphysical properties of the surface were not considered in the present model. In addition, the details of the beetle body’s geometry, including legs, core, mouth etc., as well as the skin tessellation, wettability, etc. are properties that may also influence the effectiveness of water capture (in addition to head stance) and, indeed, represent interesting avenues for future follow-up studies. These properties could also inform possible strategies to develop materials and geometries that mimic the beetle’s fog-basking characteristics.

## Figures and Tables

**Table 1:** Parameters of the simulations. The computational domain has $N=2.5\times 10^{6}$ grid points. Volume fraction $f_{p}$ refers to droplets homogeneously distributed over a slab extending $L_{\mathrm{slab}}=4.7\;\mathrm{mm}$ in the streamwise direction. Free-stream temperature is fixed at $T_{0}=285\;K$, characteristic of actual conditions of fog basking in the Namib desert. For runs with droplets of uniform size, $d_{p}=20\;\mu m$; for runs with droplets following the Rosin-Rammler distribution (Equation (8)), ${\bar{d}}_{p}=23.48\;\mu m$ and n=4.32.

Run	Inclination Angle	Free-Stream Velocity	# Droplets	Volume Fraction	Size Distribution
	α (deg)	$U_{0}\;\left(m/s\right)$	$N_{p}$	$f_{p}$	
A1–A10	0,10,20,30,35,40,45,50,55,60	1	$3.43\times 10^{5}$	$3.6\times 10^{-3}$	Uniform
B1–B10	\\	2.7	\\	\\	\\
C1–C10	\\	5	\\	\\	\\
D1–D10	\\	1	$3.43\times 10^{4}$	$3.6\times 10^{-4}$	\\
E1–E10	\\	2.7	\\	\\	\\
F1–F10	\\	5	\\	\\	\\
G1–G10	\\	1	$3.43\times 10^{5}$	$2.7\times 10^{-3}$	Rosin–Rammler

**Figure A1:**
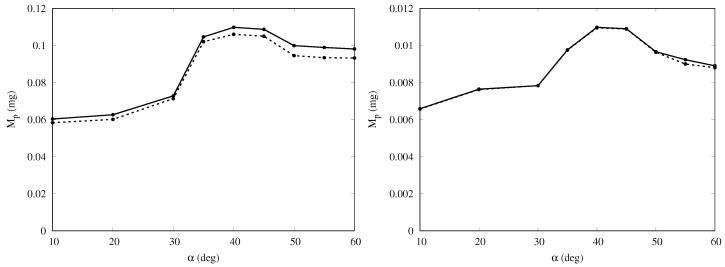
Water mass collected (as droplets) on the semi-ellipsoidal surface for varying body inclinations α and either two-way (solid lines) or one-way (dashed lines) coupling between the two phases. Free-stream velocity is $U_{0}=1\;m/s$. Left panel: $f_{p}=3.6\times 10^{-3}$; right panel: $f_{p}=3.6\times 10^{-4}$.

## Supplementary Materials

The following are available at https://www.mdpi.com/2313-7673/4/3/59/s1.

## Appendix A

We performed an additional set of simulations with the two-way coupling term $F_{D}$ in Equation (2) switched off. This corresponds to the one-way coupling assumption where the effect of droplets on the fluid motion is neglected. This assumption is often invoked in CFD in cases with low droplet volume fraction, because the simulations are computationally faster than their two-way coupled counterparts. Figure A1 reveals that the mass collected on the body follows the same trend (vs. inclination) for both one-way and two-way coupling, even though for the higher volume fraction, one-way coupling slightly underpredicts the mass collection, particularly at high inclinations. This behavior is expected because the drag force experienced by droplets carried in the fluid decelerates them and this eventually leads to a local fluid acceleration at the same droplet position as a mere consequence of the balance of forces between the two phases. The net effect is that droplets experience a higher fluid velocity, which results in a larger mass collection, as already discussed in Figure 6. Note that the peak in mass collection for the two-way coupled simulations remains at around α=40deg. These simulations suggest that future work requiring more detailed representation of the beetle complex geometry, thus necessitating more expensive simulations (larger computational domains and finer grids), could adopt one-way coupling to produce reliable predictions, as long as droplet volume fractions remain below $10^{-4}$.
