# Supplementary figures and images for: Importance of Body Stance in Fog Droplet Collection by the Namib Desert Beetle
Source: Biomimetics (Basel). 2019 Aug 28;4(3):59. doi: 10.3390/biomimetics4030059 (PMC6784302; doi:10.3390/biomimetics4030059)

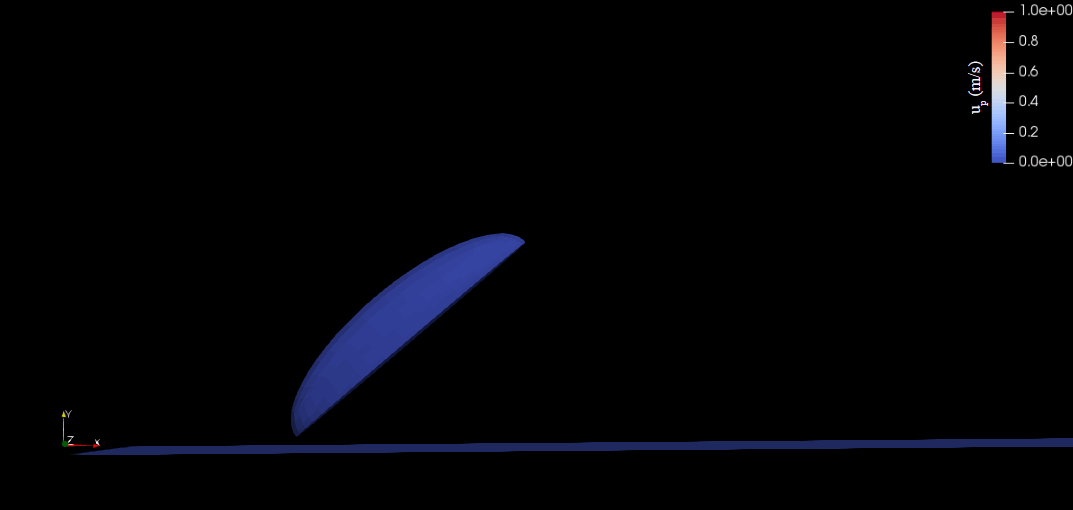

Supplement: Supplementary file 1 [file biomimetics-04-00059-s001.zip › biomimetics-527149-supplementary/fig5_animation.gif]

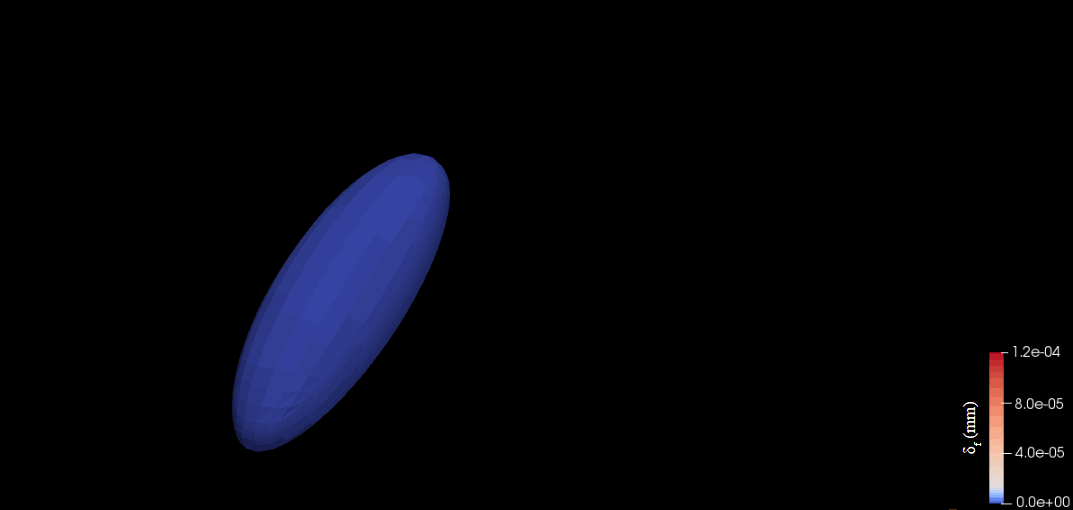

Supplement: Supplementary file 1 [file biomimetics-04-00059-s001.zip › biomimetics-527149-supplementary/fig9_animation.gif]
